# Expressions of Condolence

**Published:** 1914-06

**Authors:** 


### Expressions of Condolence.

From all over the country telegrams and letters of sympathy and condolence have been received in regard to the death of Dr. Daniel, containing expressions of love and esteem for him and expressive of appreciation of the TEXAS MEDICAL JOURNAL. All these have been greatly appreciated and have been of great encouragement. Only lack of space prevents the reproduction of many of these kind expressions, and extracts from a few are here given:

Dr. G. B. Foscue, Waco: "I have known him long and well; he was my friend and I shall miss him very much indeed. I am at your service at all times and will gladly do what I can towards contributing to the success of the TEXAS MEDICAL JOURNAL."

Dr. F. Paschal, San Antonio: "I regret very sincerely the death of your dear husband. I counted him among my best friends and feel that the profession has lost a valuable member, the country a good citizen and many a good friend. I hope that you will be able to continue conducting the JOURNAL and am glad to know that you have fitted yourself for the work."

Dr. J. T. Benbrook: "There was only one Dr. Daniel, bright, scintillating, ever ready to fight for the right; had no compromise to make with the wrong. He was aggressive, brave and dared to do right for right's sake. His place is vacant; no one can fill it just as he did.

"Truly none really knew him but to love him;
None named him but to praise."

Dr. John Preston, Austin: "Dr. Daniel was always a good friend of mine and I hope you will be successful in conducting the JOURNAL and that his many friends may assist you in every way in their power."

Dr. George H. Lee, Galveston: "I was very fond of Dr. Daniel, as you know, and admired him, and feel a personal loss in his death. I will be glad to do anything in my power to assist you in the work you have undertaken."

Dr. Bacon Saunders, Fort Worth: "I assure you of my sincere and heartfelt sympathy in your irreparable loss of my long-time friend, Dr. Daniel. Such as I can do to help you in your work will be done cheerfully."

Dr. Charles H. Hughes, St. Louis: "I thought much of the Doctor. He was a noble man, much esteemed for the probity of his character and the lofty life aims he cherished. You are offered by services in any way you may desire them."

The Denver Chemical Co.: "We are glad to know that you are to continue publishing the JOURNAL, and we wish to assure you it will be a pleasure to continue to advertise with you."

A. R. Elliott, New York: "I had the highest respect for Dr. Daniel, and he stood in very favorable light, not only in connection with my advertising agency, but also with the *New York Medical Journal,* of which I am publisher. We shall continue to use the TEXAS MEDICAL JOURNAL."

Dr. George E. Pettey, Memphis, Tenn.: "I feel the loss of Dr. Daniel very keenly. I appreciate the work your JOURNAL has been doing and feel sure that you will be able to continue it successfully. Just let our card run from year to year and mail us bills, which will be paid."

Purdue Frederick Co., New York: "We shall miss Dr. Daniel very much, as we have had more correspondence with him than any other editor we can remember, and the letters were always along cheerful lines. We are glad to know that you will continue the publication and assure you it will be our pleasure to continue one of your supporters."

Kress & Owen, New York: "We regret exceedingly to hear of the death of your husband, Dr. Daniel. We assure you that we wish you all success in your undertaking, and will extend you our hearty support."

New Orleans Polyclinic: "We shall be glad to continue our patronage of the JOURNAL, not only as a purely business transaction, but also out of regard for your lamented husband and your pluck in continuing the work in his stead."

The Norwich Pharmacal Co., Norwich, N. Y.: "We are particularly grieved to note the death of your esteemed husband, whose JOURNAL has regularly crossed the writer's desk for some years back and to congratulate you on your courage in carrying on this undertaking and to say we believe your efforts will be crowned with success."

The Temple Sanitarium, Temple, Texas: "We regret exceedingly to hear of Dr. Daniel's death, and we assure you that we all sympathize with you in your loss. We shall be glad to continue our patronage and trust you will be entirely successful in your undertaking."

Morse International Agency, New York: "We note you are to continue the issuance of the TEXAS MEDICAL JOURNAL and trust that you may have great success and that the stand for the right that has for so many years been the policy of your publication may be its future policy."

Mellier Drug Co., St. Louis: "We learn with much regret of the death of your esteemed husband, Dr. F. E. Daniel, with whom we have had such pleasant relations for so many years and whom we held in such high regard. We are pleased to continue our advertisement as heretofore with best wishes for your success."

Walter P. Long, New York: "It is with great sorrow that I learned of the death of your husband, whom though I had never met, I had come to hold in high esteem. I am glad you are to carry on the business, and wish you abundant success."

These are but a few extracts from the many letters and telegrams that have been received, all of which indicate the regret of friends and patrons at the passing of Dr. Daniel, and express kindest wishes for the continued success of the TEXAS MEDICAL JOURNAL. Such expressions inspire one to put forth the very best efforts to meet every expectation of friends.

